# Clinical Features Associated with Female Genital Mutilation/Cutting: A Pilot Longitudinal Study

**DOI:** 10.3390/jcm9082340

**Published:** 2020-07-22

**Authors:** Georgios Paslakis, Josep M. Farré, Iris Tolosa-Sola, Alba Palazón-Llecha, Helena Domínguez-Cagnon, Maria Jiménez, Berta Martínez Rosselló, Pere Barri-Soldevila, Gemma Mestre-Bach

**Affiliations:** 1 Toronto General Hospital, University Health Network, Toronto, ON M5G 2C4, Canada; 2 Department of Psychiatry, University of Toronto, Toronto, ON M5T 1R8, Canada; 3 Department of Psychiatry, Psychology and Psychosomatics, Dexeus University Hospital, 08028 Barcelona, Spain; psico.dex@quironsalud.es (J.M.F.); iris9990@gmail.com (I.T.-S.); albapalazonllecha@gmail.com (A.P.-L.); helenapsicodex@gmail.com (H.D.-C.); mjb.jimenezbonora@gmail.com (M.J.); bmartinezrossello@gmail.com (B.M.R.); 4 Department of Obstetrics, Gynecology, and Reproduction, Dexeus University Hospital, 08028 Barcelona, Spain; barper@dexeus.com; 5 Facultad de Ciencias de la Salud, Universidad Internacional de La Rioja, 26006 La Rioja, Spain

**Keywords:** female genital mutilation/cutting, body image, genital self-image, female sexual function, psychopathology, clitoral reconstruction

## Abstract

(1) Background: Female genital mutilation/cutting (FGM/C) is associated with physical and psychological complications. However, there is scarce literature on how women with FGM/C respond to treatment interventions. (2) Methods: In the present pilot longitudinal study, we assessed changes in general psychopathology (Symptom Check List-90-R), sexual functioning and distress (Female Sexual Function Index, Female Sexual Distress Scale-Revised, and Diagnostic and Statistical Manual of Mental Disorders, Fifth Edition (DSM-5) female sexual dysfunction criteria), body image (Body Shape Questionnaire), and sexual body image (Female Genital Self-Image Scale) in a sample of *n* = 15 women with FGM/C before and after reconstructive surgery. (3) Results: Sexual distress was significantly improved following surgery. We also observed an improvement in general psychopathology and genital self-image. However, sexual function was not improved. (4) Conclusions: These results provide evidence for the benefits of reconstructive surgery on sexual distress in women with FGM/C. The impact of surgery on sexual function cannot be conclusively evaluated.

## 1. Introduction

The World Health Organization has defined female genital mutilation/cutting (FGM/C) as any intentional procedure to partly or entirely remove external female genitalia, or any injury to the female genitalia that is non-therapeutic [1]. FGM/C has been classified into four different types depending on the extent to which genital tissue is removed. FGM/C is a deeply rooted cultural and still ongoing practice in a significant number of countries across Africa, the Middle East, Asia, and the Pacific. However, with migration, it has spread to countries like Spain. In Spain, there are currently different scenarios to consider: (1) girls or adult women who already arrive in the country with FGM/C; (2) girls born in Spain, who undergo FGM/C during a holiday or long-term trip to their parents’ country of origin, regardless of parental consent to the procedure; and (3) girls born in Spain, who undergo FGM/C on Spanish territory or in other European countries, although there are no records of this scenario since the 1990s [2].

FGM/C is performed for different reasons, but usually as a ritual representing the shift to womanhood and obtaining legitimacy as new member of the community [2,3,4]. Nonetheless, it is considered a violation of human rights of the affected individuals [1] and, in Europe, several initiatives and policies have been formed to address it [5].

From a sexual medicine perspective, women who suffered FGM/C often show a burden of short- and long-term gynecological and obstetric impairments, including urinary retention, genital scarring, infections, bacterial vaginosis, perinatal complications and menstrual difficulties [6,7,8,9,10,11,12,13]. Relatedly, FGM/C is also associated with sexual function impairments [10,14]. A reduction in sexual satisfaction, along with less sexual activity and sensitivity, and female sexual dysfunctions, especially genito-pelvic pain/penetration and female orgasmic disorders have been reported by women with FGM/C [15,16].

Although many authors uphold the negative sexual and physical impact of FGM/C, research focused on the correlates of psychopathology and FGM/C is still in its nascent stages [17]. Most commonly identified manifestations of psychological impairments include negative self-image, depression, anxiety, somatization, and posttraumatic stress disorder [17,18,19,20,21,22,23,24]. In a recent comprehensive literature review, 14 of the 16 included studies reported an association between FGM/C and at least one adverse mental health outcome [25].

Different options for reconstruction of the female genital after FGM/C have been proposed, such as defibulation, removal of cysts, reconstruction of clitoris and labia, neuromas, and scar tissue, although clitoral reconstruction (CR) has been the most widely used [24,26]. CR has been shown to improve both genital anatomy and sexual function, including favorable changes in genital self-image [10,27,28,29,30]. There is some evidence showing that CR may also improve gynecologic and obstetric outcomes [31], although it may not improve urological complications [32]. However, the impact of CRon the psychological and sexual well-being of women with FGM/C is largely uninvestigated. Preliminary studies in this area have reported reduced depression and sexual distress levels and possible improvements in body image after CR [16,24,33]. However, it appears that genital self-image is not significantly improved after CR [16]. More studies are needed as genital self-image has been shown to be associated with sexual function [34,35,36].

Several barriers related to economic and (infra-)structural aspects (e.g., costs or the distance to specialized health care facilities), socio-cultural factors (e.g., cultural taboos), concerns about the quality of care, and fears of legal sanctions have been identified to limit health care utilization by women who have suffered FGM/C [37]. Furthermore, the perception of one’s genital image as “ugly” due to FGM/C may also lead affected women to abstain from seeking health care support such as gynecological screenings [38].

In the present study, a series of sexual and psychopathological variables including body image and genital self-image were assessed in women who had suffered FGM/C. The aim of the study was to re-evaluate sexual function and psychological well-being in these women after they had undergone CR. Our hypothesis was that CR would lead to an amelioration in all aspects under investigation.

## 2. Experimental Section

### 2.1. Participants and Procedure

The present longitudinal study included *n* = 43 women with Type I and II FGM/C who sought CR at the Department of Obstetrics, Gynecology, and Reproduction at our hospital. The study was conducted in a private hospital, located in an urban area of Spain, although the entire intervention was covered by a charitable foundation, meaning patients did not have to incur any financial costs. All participants included in the study were living in Spain and were fluent in Spanish. They were referred by their primary health care physicians or sought treatment themselves. All were consecutive referrals for assessment and treatment between March 2015 and January 2020.

For this study, FGM/C types were categorized according to the WHO classification by a gynecologist who is an expert in this field [1]. Type I, also known as clitoridectomy, refers to the partial or total removal of the clitoris, and only in some cases, only the removal of the prepuce. Type II, also known as excision, includes the partial or total removal of the clitoris and the labia minora, with or without excision of the labia majora.

Inclusion criteria were to be women, between the ages of 18 and 45 (except for two cases outside this age range who underwent reconstructive surgery based on the clinical indication set by gynecologists), and being sexually active (to determine the presence of sexual dysfunctions or distress). Exclusion criteria were having an intellectual disability or severe mental disorder, being pregnant, and having a mutilation considered to not be eligible for CR. Patients who met inclusion criteria were assigned to a therapist at the Department of Psychiatry, Psychology and Psychosomatics at our hospital and were considered potential participants for this study.

**Figure 1:**
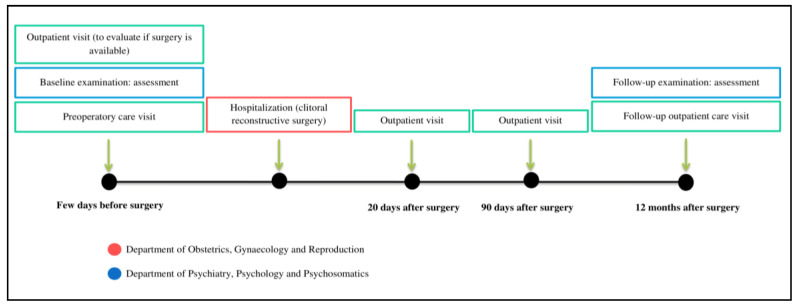
Clinical procedure timeline.

All participants were assessed in a face-to-face clinical interview by a clinical psychologist before CR and a total of n=15 participants were also assessed 1 year after surgery. The remaining patients did not attend the follow-up session after one year post-CR. Sociodemographic and clinical information was assessed at baseline and the 1-year follow-up, as required. Patients also individually completed all questionnaires (requiring approximately 40 min) at these two time points (Figure 1 includes the timeline of the present study) (see Figure 1).

### 2.2. CR Procedure

CR consists of uncovering the remaining clitoris and placing it externally as close to the vagina as possible. The procedure consists of first removing scar tissue from the skin to expose the clitoris. When the residual clitoris is recognized, the clitoris is dissected from bulbocavernosus muscles in order to obtain lateral mobility and the suspensory ligament is sectioned. The anchorage of the gland is performed with Vycril sutures that encroach both the pubic periosteum and the ventral base of the clitoris. The skin is closed with interrupted 3–0 stitches. Patients are discharged one day after surgery and are required to attend checkup visits. The patient is instructed on how to perform daily wound care the first month following surgery. Sexual intercourse is allowed 3 months after surgery and a final evaluation is made at 1 year after CR.

### 2.3. Measures

#### 2.3.1. Psychopathology: Symptom Check List-90-R (SCL-90-R) 

This 90-item questionnaire featuring a five-point Likert scale format was used to evaluate nine symptom dimensions (somatization, obsession-compulsion, interpersonal sensitivity, depression, anxiety, hostility, phobic anxiety, paranoid ideation, and psychoticism) and three global indices (distress, severity, and positive symptoms) [39]. The Spanish validation has shown adequate internal consistency (0.76 and 0.98) [40].

#### 2.3.2. Sexual Functioning: Female Sexual Function Index (FSFI) 

The FSFI [41] is a 19-item self-report measure which assesses sexual function in females. It is made up of six domains: desire, arousal, lubrication, orgasm, satisfaction, and pain. It has been shown to have good psychometric properties and clinical utility. Next to the individual domains, a total score is also calculated. The Spanish version was validated by Blumel et al. [42]. Sexual dysfunction was assessed by adding the scores from the different domains of the FSFI.

#### 2.3.3. Sexual Distress: Female Sexual Distress Scale-Revised (FSDS-R) 

The FSDS-R [43] is a 13-item questionnaire which assesses different components of sexual distress in women over the last 4 weeks. Items on the FSDS-R are scored using a five-point Likert-type scale (never (0), rarely (1), occasionally (2), frequently (3), or always (4)) and provide a total score; higher scores indicate higher levels of sexual distress. The original version has demonstrated adequate reliability (α =0.87 to α = 0.93) and high test–retest reliability (*r* = 0.74 to *r* = 0.86) [43].

#### 2.3.4. Sexual Functioning: Diagnostic and Statistical Manual of Mental Disorders (DSM-5) 

DSM-5 female sexual dysfunction criteria were used in the present study [44]. The DSM-5 recognizes the following disorders as female sexual dysfunctions: female sexual interest or arousal disorder (mainly characterized by absent or reduced interest in sexual activity, sexual or erotic thoughts or fantasies, and sexual excitement or pleasure during sexual activity or to any internal or external sexual or erotic cues); female orgasmic disorder (characterized by a marked delay in, infrequency, or absence of orgasm and/or reduced intensity of orgasmic sensations); and genito-pelvic pain or penetration disorder (mainly difficulties in vaginal penetration or vulvovaginal or pelvic pain during intercourse).

#### 2.3.5. Body Shape: Body Shape Questionnaire (BSQ) 

The BSQ [45] evaluates body dissatisfaction related to body self-perception. It comprises 34 items with six possible responses and it explores four factors: body dissatisfaction, fear of gaining weight, feelings of low self-esteem related to appearance, and desire to lose weight. A total score is calculated. BSQ has shown high reliability (Cronbach’s alpha between 0.95 and 0.97). The adapted version to Spanish population was used in the present study [46].

#### 2.3.6. Sexual Body Image: Female Genital Self-Image scale (FGSI) 

This is a reliable and valid measure to assess female genital self-image [47]. It consists of seven items related to women’s feelings and beliefs about their own genitals, and it uses a 4-point response scale: strongly agree, agree, disagree, and strongly disagree. A total score is calculated. The scale was found to have adequate reliability (α = 0.88). Moreover, questionnaire scores were found to be positively associated to sexual function [47].

#### 2.3.7. Davidson Trauma Scale (DTS) 

The DTS [48] is a 17-item self-report instrument. It is used to assess the 17 DSM-IV symptoms of post-traumatic stress disorder. Items are rated on 5-points frequency (0 “not at all” to 4 “every day”) and severity scales (0 “not at all distressing” to 4 “extremely distressing”). A cut-off score of 40 demonstrated a sensitivity of 0.69 and a specificity of 0.95 [48].

#### 2.3.8. Other Sociodemographic, Sexual and Clinical Variables

Sociodemographic variables, sexual, and psychiatric history were assessed by means of a semi-structured face-to-face clinical interview.

### 2.4. Statistical Analyses

All statistical analyses were performed using SPSS (IBM SPSS Statistics for Windows, Armonk, NY: IBM Corp.). Appropriate descriptive statistics were calculated for all sociodemographics, sexual and psychiatric history, DSM-5 sexual disorders, mutilation type, and all questionnaires applied at baseline. Paired T-Tests were performed to investigate changes in all measures under investigation from baseline to follow-up (1 year postoperatively). The level of significance was set at *p* ≤ 0.05.

### 2.5. Ethics

The present study was carried out in accordance with the latest version of the Declaration of Helsinki. The Hospital Clinical Research Ethics Committee approved the study, and signed informed consent was obtained from all participants.

## 3. Results

### 3.1. Demographics

Data from a total of *n* = 43 women with FGM/C and a mean age of 27.88 ± 7.33 years were analyzed in this study. One patient underwent reconstructive surgery at age 17 and another one at age 48 based on clinical (gynecological) judgment. Of these women, *n* = 41 had suffered a type II FGM/C, and *n* = 2 a type I FGM/C. Place of birth was West Africa in most (*n* = 30) of cases. The majority of these women were single (*n* = 26, 60.5%) and had no children (*n* = 26, 60.5%). Most of them had high school education (*n* = 26, 60.5%) and were employed at the time of study participation (*n* = 29, 67.4%). The vast majority of women also had no previous psychiatric history (*n* = 38, 88.4%). Demographics are shown in Supplementary Table S1.

Of these *n* = 43 women and *n* = 15 were re-assessed post-operatively. Their mean age was 28.80 ± 7.41 years. Most of these women (73.3%) were single and had no children (73.3%). No psychiatric history was reported in 86.7% (*n* = 13) of the women. Demographics of the women who took part in the longitudinal investigation are presented in Table 1.

### 3.2. Baseline Measures

Based on clinical interviews to assess sexual disorders according to the DSM-5, more than half of the cohort fulfilled the criteria for sexual interest/arousal disorder (*n* = 25, 58.1%), and even more reported symptoms of an orgasmic disorder (*n* = 30, 69.8%). However, the percentage of women with genito-pelvic pain or penetration disorder (*n* = 17, 39.5%) was lower than the percentage of women who denied symptoms of this kind.

Results were similar among women in the longitudinal cohort; sexual interest/arousal disorder and orgasmic disorder were again the most prevalent symptoms (*n* = 8, 53.3% and *n* = 10, 66.7%, respectively), while *n* = 5 women reported genito-pelvic pain or penetration disorder (33.3%) (see Table 2).

The rest of the scores obtained for *n* = 43 women during the pre-surgical evaluation are displayed in supplementary Table S2.

### 3.3. Post-Operative Measures

Only *n* = 15 women returned to our unit one year after the CR. We attempted to contact women and ask for the reasons for refraining from their re-assessment appointments. Reasons were mainly having moved to another country for work or due to family issues or having found a job and not being able to accommodate medical visits.

In the group of women who were re-examined, psychological well-being, as measured by the SCL-90-R, was significantly improved in five of the nine subdomains (somatization, interpersonal sensitivity, depression, phobic anxiety, and paranoid ideation). All global indices of psychological distress were also significantly improved (see Table 3).

There was a highly significant improvement in FSDS-R scores as a measure for sexual distress (*p* < 0.001; see Table 3). However, all sexual function subscales of the FSFI, including sexual satisfaction, did not significantly improve (see Table 3).

Finally, while body shape concerns were not significantly different during the second assessment, there was a significant improvement in genital self-image (*p* = 0.02) (see Table 3).

Effect sizes for significant differences ranged from 0.56 to 1.22 (Cohen’s d), the majority being medium effect sizes with the exception of sexual distress (Cohen’s d = 1.22) (Table 3).

## 4. Discussion

The present study analyzed whether sexual function, sexual distress, body image and genital self-image, as well as general psychopathology in women with FGM/C improved after CR. We hypothesized that all of the assessed measures would significantly improve following surgery. Our hypotheses were partially supported.

As a main finding, a significant decrease in sexual distress after CR was noted (*p* < 0.001, Cohen’s d = 1.22). Thus, the highly statistically significant difference observed with regard to this aspect may be considered as evidence for the benefits of CR in women with FGM/C. On the other hand, sexual function did not improve. Despite a reduction in all FSFI scores, suggesting an improvement in sexual function after genital reconstruction, the differences were not statistically significant. This could either be cautiously interpreted as an indication that sexual distress is significantly improved regardless of recovery of sexual function or may be attributed to the small number of women assessed at follow-up (underpowered sample to detect significant changes in sexual function). Previous evidence on sexual function after CR is limited overall. However, some studies in this area suggest that CR might be beneficial for improving sexual function and pain [28,29,49,50]. A previous study in 12 women with FGM/C using the FSFI found an improvement in all dimensions except lubrication [10]. Interestingly, the mean total score in the post evaluation in that study was >23 [10], similar to the one we obtained here (23.84). Therefore, the authors concluded that FSFI is a suitable psychometric tool for conducting a standardized assessment of sexual function in women with FGM/C. A recent systematic review, which has included 62 studies (5829 women), has reported that up to 22% of women with FGM/C showed worsening of sexual function after CR [51].

Previous studies hypothesized that women’s general perception of their bodies may extend to different parts of the body, such as the genitals [52,53]. To examine inferences between body shape perception and genital self-image, both aspects were simultaneously assessed in the present study. A non-significant increase in satisfaction with body shape and a significant improvement in genital self-image were observed after CR. CR directly affects the genitals; it is therefore not surprising that there is an improvement in genital self-perception. At the same time, our finding points towards a lack of interdependence of the two concepts: improvement in genital self-image does not go along with an impact on body image perception. Again, the small cohort of this pilot study at follow-up should be considered while interpreting these results. To the best of our knowledge, only one previous case study assessed female genital self-image in a woman with FGM/C, showing a worsening in clitoral self-image after CR [16]. Previous studies assessing female genital self-image have observed that the dissatisfaction with one’s genitals may reduce the frequency of gynecological examinations [54]. In light of our finding and the critical observation by Demaria et al. [54], CR may contribute to better health care, which should be taken into account by health professionals providing care in women with FGM/C.

Finally, a significant overall improvement was evident when comparing general psychopathology features in women with FGM/C before and after CR (medium effect sizes Cohen’s d), although this improvement cannot be causally attributed to the surgical intervention.

Overall, as it is not yet clear if the intended benefits of CR outweigh associated risks [55], caution should be applied in interpreting the present findings.

### Strengths, Limitations and Future Research

This study longitudinally evaluated a series of clinical aspects in women with FGM/C using validated psychometric tools. Our pilot study contributes to a further understanding of how CR might influence the well-being of these patients.

However, there are some drawbacks that should be highlighted. Our sample size was small, thus has limiting statistical power. Attrition to follow ups is a relevant limitation in studies of the kind. Future studies would be able to draw more specific conclusions by including large sample sizes and controlling for confounding variables. It remains unclear which individual and socio-cultural factors might be limiting adherence to medical follow-ups; tailored plans to prevent attrition need to be implemented. All data used in this study were collected from individuals who were voluntarily attending treatment and agreed to participate. Hence, the generalizability of our results to the entire population of women with FGM/C is limited. Additionally, despite our attempt to use a combined method of clinical interviewing and self-report to overcome biases in the evaluation, both procedures could be subject to a high degree of social and cultural desirability bias. Finally, a post-operative clinical interview based on the DSM-5 criteria could not be conducted in all patients, which should be considered a relevant limitation of the study.

## 5. Conclusions

Taken together, there are potential benefits of CR regarding sexual distress and genital self-image in women with FGM/C. However, the risks of the intervention must be taken into account and further research in this field is needed to determine the most adequate and effective interventions for women with FGM/C.

## Figures and Tables

**Table 1:** Sample characteristics.

Country of Birth	N	%
Spain	3	21.5
Gambia	1	7.2
Ethiopia	1	7.2
Mali	2	14.2
Guinea	3	21.5
Senegal	2	14.2
Ivory Coast	2	14.2
Total	14	100
**Family Status**	**N**	**%**
single	11	73.3
married/relationship	3	20.0
separated	1	6.7
Total	15	100
**Children**	**N**	**%**
none	11	73.3
1	2	13.3
2	2	13.3
Total	15	100
**Education**	**N**	**%**
elementary school	2	13.3
high school	7	46.7
higher education	6	40.0
Total	15	100
**Occupation**	**N**	**%**
employed	10	66.7
student	2	13.3
unemployed	3	20.0
Total	15	100
**Current Sexual Activity**	**N**	**%**
yes	8	61.5
no	5	38.5
Total	13	100
**Mutilation type**	**N**	**%**
Type I	2	13.3
Type II	13	86.7
Total	15	100.0
**Psychiatric History**	**N**	**%**
none	13	86.7
anxiety	1	6.7
other	1	6.7
Total	15	100

**Table 2:** Rates of DSM-5 sexual disorders among women in the longitudinal cohort.

DSM-5 Disorders		
**Sexual interest or arousal disorder**	**N**	**%**
Yes	8	53.3
No	7	46.7
Total	15	100.0
**Orgasmic disorder**	**N**	**%**
Yes	10	66.7
No	5	33.3
Total	15	100.0
**Genito-pelvic pain or penetration disorder**	**N**	**%**
Yes	5	33.3
No	10	66.7
Total	15	100.0

**Table 3:** Comparison between baseline measures (pre) and post-operative measures (one year, post) with regard to psychological well-being, sexual distress, sexual function, body image and genital self-image in women who suffered FGM/C.

Questionnaires	N	Mean	Std. Deviation	P	Cohen’s d
**SCL-90-R**					
SCL Somatization pre	15	1.21	0.91		
post	15	0.82	0.82	**0.02**	0.68
SCL Obsessive Compulsive pre	15	1.79	1.07		
post	15	1.25	0.92	0.11	0.44
SCL Interpersonal Sensitivity pre	15	1.58	1.12		
post	15	0.96	0.90	**0.01**	0.74
SCL Depression pre	15	1.63	1.19		
post	15	1.05	1.07	**0.05**	0.56
SCL Anxiety pre	15	1.23	1.06		
post	15	0.83	0.96	0.14	0.40
SCL Hostility pre	15	1.02	1.06		
post	15	0.66	1.02	0.19	0.35
SCL Phobic Anxiety pre	15	0.98	1.23		
post	15	0.43	0.68	**0.02**	0.71
SCL Paranoid Ideation pre	15	1.56	1.13		
post	15	0.92	1.11	**0.03**	0.63
SCL Psychoticism pre	15	1.18	1.10		
post	15	0.70	0.95	0.08	0.49
SCL Global Severity Index pre	15	1.43	0.93		
post	15	0.90	0.86	**0.02**	0.69
SCL Positive Symptom Total pre	15	49.13	25.46		
post	15	35.73	24.87	**0.04**	0.58
SCL Positive Symptom Distress Index pre	15	2.26	0.80		
post	15	1.83	0.81	**0.02**	0.65
**FSDS-R**					
FSDS-R pre	15	28.93	14.44		
post	15	12.93	13.30	**p < 0.001**	1.22
**FSFI**					
FSFI Desire pre	15	4.63	2.62		
post	15	4.36	1.77	0.74	0.09
FSFI Arousal pre	15	5.39	5.20		
post	15	4.20	4.83	0.49	0.18
FSFI Lubrication pre	15	7.26	7.77		
post	15	3.88	5.01	0.14	0.40
FSFI Orgasm pre	15	3.89	4.14		
post	15	2.96	3.87	0.38	0.24
FSFI Satisfaction pre	15	4.61	3.47		
post	15	4.35	3.29	0.78	0.08
FSFI Pain pre	15	4.11	5.33		
post	15	4.03	3.68	0.95	0.02
FSFI Total pre	15	29.16	23.79		
post	15	23.84	21.57	0.44	0.21
**BSQ**					
BSQ pre	15	93.67	48.54		
post	15	81.33	57.98	0.20	0.35
**FGSI**					
FGSI pre	15	14.27	5.35		
post	15	20.40	6.45	0.02	−0.71

## Supplementary Materials

The following are available online at https://www.mdpi.com/2077-0383/9/8/2340/s1, Table S1: Sample characteristics, Table S2: Baseline measures of psychological well-being, depression, anxiety, sexual distress, sexual function, body image, genital self-image, and trauma scores in women with FGM/C.
