# Supplementary material for: Clinical Features Associated with Female Genital Mutilation/Cutting: A Pilot Longitudinal Study
Source: J Clin Med. 2020 Jul 22;9(8):2340. doi: 10.3390/jcm9082340 (PMC7463820; doi:10.3390/jcm9082340)
Supplement: Supplementary file 1 [file jcm-09-02340-s001.pdf]

Supplementary Table S1. Sample characteristics.

Country of Birth	N	%
Spain	10	23.3
Portugal	1	2.3
Gambia	7	16.3
Ethiopia	1	2.3
Mali	6	14.0
Guinea	7	16.3
Senegal	8	18.6
Ivory Coast	2	4.7
Total	42	100
**Family Status**	**N**	**%**
single	26	60.5
married/relationship	11	25.6
widowed	2	4.7
separated	3	7.0
Total	42	100
**Children**	**N**	**%**
none	26	60.5
1	6	14.0
2	6	14.0
3	4	9.3
Total	42	100
**Education**	**N**	**%**
elementary school	4	9.3
high school	26	60.5
higher education	10	23.3
Total	40	100
**Occupation**	**N**	**%**
employed	29	67.4
student	7	16.3
unemployed	6	13.9
Total	42	100
**Current Sexual Activity**	**N**	**%**
yes	22	66.7
no	11	33.3
Total	33	100
**Mutilation type**	N	%
Type I	2	4.65
Type II	41	95.35
Total	43	100.0
**Psychiatric History**	**N**	**%**
none	38	90.4
anxiety	1	2.4
depression	2	4.8
other	1	2.4
Total	42	100

Supplementary Table S2. Baseline measures of psychological well-being, depression, anxiety, sexual distress, sexual function, body image, genital self-image, and trauma scores in women with FGM/C.

Questionnaire	N	mean	Std. deviation
**SCL-90-R**			
SCL Somatization	43	0.93	0.84
SCL Obsessive Compulsive	43	1.50	0.93
SCL Interpersonal Sensitivity	43	1.27	0.93
SCL Depression	43	1.33	1.04
SCL Anxiety	43	0.99	0.97
SCL Hostility	43	0.85	0.84
SCL Phobic anxiety	43	0.64	0.94
SCL Paranoid Ideation	43	1.31	0.98
SCL Psychoticism	43	0.89	0.88
SCL Global Severity Index	43	1.12	0.81
SCL Positive Symptom Total	43	41.67	23.04
SCL Positive Symptom Distress Index	43	2.17	0.73
**FSDS-R**	42	31.14	13.36
**FSFI**			
FSFI Desire	40	3.45	2.28
FSFI Arousal	40	3.41	3.99
FSFI Lubrication	40	4.67	5.83
FSFI Orgasm	40	2.84	3.26
FSFI Satisfaction	40	3.78	2.57
FSFI Pain	40	3.54	4.11
FSFI Total	40	22.78	23.03
**BSQ**	43	74.40	45.04
**FGSI**	43	14.77	4.33
**DTS**			
DTS Trauma	24	2.96	1.94
DTS Trauma frequency	38	45.45	159.62
DTS Trauma severity	38	46.47	159.67
DTS Trauma total	38	65.63	159.10
